# Experimental Investigation of the Pumping of a Model-Concrete through Pipes

**DOI:** 10.3390/ma13051161

**Published:** 2020-03-05

**Authors:** Martin A. Haustein, Moritz N. Kluwe, Rüdiger Schwarze

**Affiliations:** Institute of Mechanics and Fluid Dynamics, TU Bergakademie Freiberg, 09599 Freiberg, Germany; kluwe@mailserver.tu-freiberg.de (M.N.K.); ruediger.schwarze@imfd.tu-freiberg.de (R.S.)

**Keywords:** concrete, particle image velocimetry, refractive index matching, rheology, migration, segregation, pressure-loss, measurement methods

## Abstract

Many practical aspects of processing fresh concrete depend on its rheology, such as the pumping of the material. It is known that a lubricating layer is formed in the process, which significantly reduces the pumping pressure. However, these phenomena can hardly be considered in the usual rheological measurements. A main problem is the optical inaccessibility of the material, which prevents estimations about, e.g., the thickness of the plug flow or particle migration. In this paper, the pneumatic pumping of a transparent model concrete is performed by means of a test plant. The flow profile over the entire pipe cross-section is resolved in time and space via Particle Image Velocimetry (PIV) measurements. This allows the comparison with the analytical flow profile from rheological measurements of the material using the Buckingham–Reiner equation. A reduction of the pressure loss to around 60% induced through segregation of the material is found. These measurements reflect the rheology of the material under realistic pumping conditions including particle migration. This makes it possible for the first time to observe a transparent material with concrete-like rheology under pulsating pumping conditions and to compare the true and calculated time-resolved pressure loss.

## 1. Introduction

Concrete is one of the most frequently used building materials worldwide. This substance enables us to construct state-of-the-art buildings and technologically sophisticated structures such as bridges and dams. One of the material’s key properties is its ability to be processed in its liquid state first and obtain a high-strength material afterwards. In the last few years, the development of new processing technologies like 3D-printing has led to a high interest in predicting the concrete’s rheological properties [1,2]. For an overview of research in this area, please refer to the excellent review by Ghaffar et al. [3,4].

At the construction site, concrete is usually transported to its destination by means of double piston pumps [5]. Unlike continuous pumping, the material is pumped at a non-constant speed. A time-dependent, pulsating flow velocity is generated, which keeps the concrete in a permanent state of acceleration and deceleration.

Concrete is a highly complex material consisting of a number of individual components, mainly cement, water, sand, and coarse aggregates, as well as chemical additives to influence the rheology to the desired extent [6,7,8]. The description of the rheology of concrete is an important, though little understood problem. A major problem is the different behavior of the different types of concrete. By using aggregates or by varying the mixing process over time, it is possible to observe flow characteristics that sometimes deviate significantly from each other [9,10,11,12,13].

Another well-known phenomenon that leads to drastic changes in the flow behavior is the segregation of the coarse material during the transport process [14,15]. Especially the segregation near the pipe wall is of great importance for the pumping of fresh concrete. The coarse aggregates migrate to the center of the pipe, while fine particles such as sand and cement paste remain on the pipe wall. A so-called lubricating layer is formed, which leads to an inhomogeneous material, but also reduces the pumping pressure [16].

The formation of the lubricating layer has been frequently investigated in recent years, for example by ultrasonic velocimetry [17], pressure loss measurements [15,18,19], and Particle Image Velocimetry (PIV) on the surface of the flowing material [20]. In addition, the influence on the pressure loss has been investigated by numerical simulation [21]. However, the exact mechanism leading to the formation of the lubricating layer is still the subject of ongoing research. Currently, the shear-induced particle migration is assumed to have a major influence [22].

One of the biggest problems in the investigation of concrete is the poor optical accessibility of the material. Because of this, many assumptions have to be made for rheological measurements. For example, a fluid with yield stress will not be sheared over the entire range. The estimation of the thickness of the shear layer and the plug-flow is error-prone. In addition, the flow behavior under pumping conditions is different from the behavior in the rheometer. To solve these problems, a transparent model concrete was employed, whose basic rheological properties were comparable to those of real concrete.

In this paper, the optical and rheological investigation of such a model concrete flowing through a pipe at a pulsating velocity is described. Due to the optical accessibility, it is possible to determine temporally and spatially resolved velocity data using PIV and thus to describe the flow profile during the pumping process. The flow profile obtained in this way contains system-relevant mechanisms such as the migration of particles. These phenomena can hardly be considered in a rheometer. Furthermore, in the present case, no assumptions about the thickness of the sheared zone or the plug-flow have to be made. The resulting data reflect the flow behavior under realistic pumping conditions. By using the Buckingham–Reiner equation, the resulting flow profile can be compared with the theoretical values obtained by rheological measurements. The influence of segregation on pump pressure and pressure drop is thus clearly shown. For the first time, the pressure loss and flow profile of a non-Newtonian, densely packed granular material under pulsating pumping conditions can be correlated with rheological data. These findings provide valuable information for the practical application of pumping concrete and for granular materials in general.

## 2. Materials and Methods

### 2.1. Plant Setup

**Figure 1:**
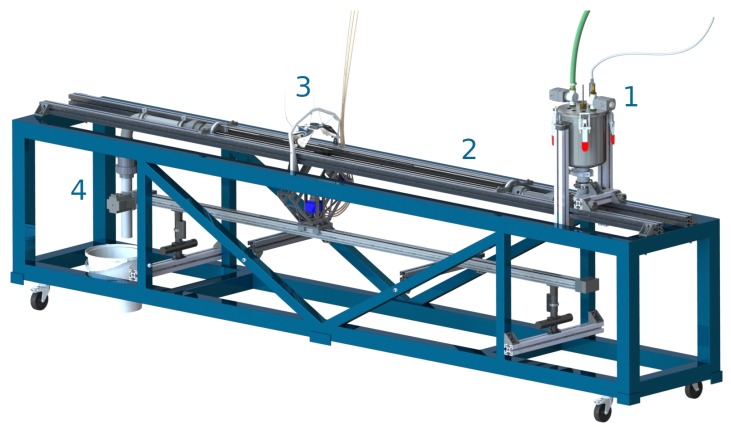
Pulsating Concrete Pump (PulsaCoP) apparatus for optical investigation of dense, granular suspensions during a pulsating flow. (1) Reservoir vessel with pressure supply and compressed-air hose. (2) Measurement pipe. (3) Camera system. (4) Outlet.

For the optical investigation of the model concrete under realistic pumping conditions, it was necessary to develop an experimental setup that allowed, on the one hand, the optical investigation of the material and, on the other hand, a time-dependent pumping process. Furthermore, the pressure loss in the system should be detectable as accurately as possible. This setup was called PulsaCoP (Pulsating Concrete Pump, TU Bergakademie Freiberg, Freiberg, Germany); see Figure 1.

**Figure 2:**
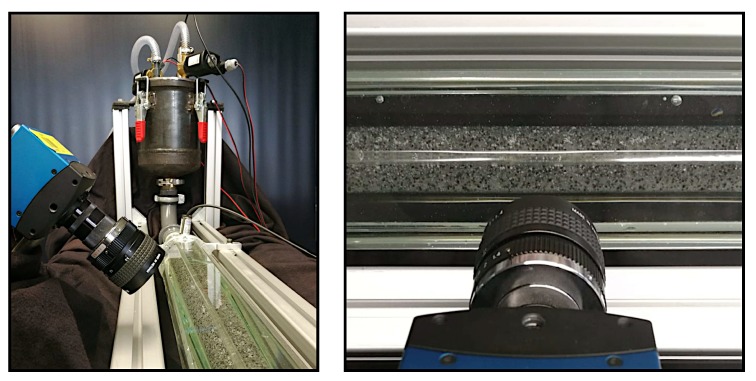
Camera setup (**left**) and close up of the model-fluid (**right**). The black cloths are used to reduce scattering.

It consisted of a reservoir that could contain up to five liters of fluid. From this reservoir, the material flowed through an elbow into the measuring section, which had a diameter of 20 mm, a length of 1470 mm, and a thickness of 2 mm. The material then flowed through another elbow and through a ball valve into a container. The measurement pipe is further depicted in Figure 2. Further details about the apparatus can be found in [2].

The model concrete was pumped pneumatically in the PulsaCoP apparatus. The pressure in the reservoir could be oscillated in a sinusoidal manner by selectively introducing and blowing out compressed air with the help of three solenoid valves attached to the reservoir. In this way, the pumping process could be simulated similar to the double piston pumps commonly used for concrete.

The heart of the system was a cylindrical pipe made of borosilicate glass. The pipe was enclosed by an outer channel with an octagonal cross-section, filled with glycerin. The refractive index of all materials: glycerin, borosilicate glass, and the model concrete were nearly identical in order to allow undisturbed optical access to the whole flow domain. Due to the adjustment of the refractive index inside and outside the curved glass tube, there was no distortion of the image. All measurements were carried out at room temperature of about 20 ${}^{\circ }C$. No influence on the measurement results was expected in the range of the usual temperature fluctuations.

The camera was a MIKROTRON Motion BLITZ EoSens mini2 (Mikrotron GmbH, Unterschleissheim, Germany) with a resolution of 15 μm per pixel. The illumination of the system was done in transmitted light setup. The images were taken at a frame rate of 100 Hz to 525 Hz. The illumination time for all measurements was 10 μs. By adjusting the focus plane, the measurement was done in the center-plane of the tube only.

The measurement setup included three independent pressure sensors. One of them was located in the storage vessel and recorded the reservoir pressure $p_{0}$. This pressure was used to control the time modulation and to regulate the opening and closing of the magnetic valves. Two further sensors $p_{1}$ and $p_{2}$ were located directly in front of and behind the measuring section. This way, the pressure loss $\Delta p=p_{2}-p_{1}$ between two defined points could be determined.

### 2.2. Model Concrete

For the investigation, a model concrete is employed whose principal rheological properties are comparable to those of concrete. It consists of borosilicate glass particles and a Bingham liquid based on a 1% Carbomer solution in water. Additional additives are used to match the refractive index of all constituents. Details of the model concrete and its preparation will be published elsewhere [23].

Both the viscosity and the yield stress could be tuned with the Carbomer concentration with respect to the values of these parameters for real concrete. The model concrete did not show any time dependency of the rheology, thus the material showed neither rheopexy nor thixotropy. To observe the behavior of the individual particles in the fluid, the glass beads and the liquid must have the same refractive index. For borosilicate glass, this index was $n_{D}$ = 1.4730.

The borosilicate glass spheres in the experiment presented here had a diameter of 0.5 mm to 1.0 mm and were produced by Schäfer-Glas. In order to obtain the densest possible packing, 70% of the 1.0 mm beads were mixed with 30% of the 0.5 mm beads. Afterwards, enough of the liquid was added to give a final solid content of $\phi_{p}=0.42$. This solid content corresponded to the proportion of solids in real concrete. Some of the larger particles were colorized for easier particle tracking.

The rheological data were determined in a Haake Mars II (building material cell) rheometer. The flow curve obtained was fitted with the Bingham model:

(1) $$\begin{array}{c} \tau =\tau_{0}+\eta_{eff}\dot{\gamma } \end{array}$$

with the yield stress $\tau_{0}$ and the effective viscosity $\eta_{eff}$. The result was a value of $\eta_{eff}=3.51\mathrm{Pa}s$ for the apparent viscosity and $\tau_{0}=9.99\mathrm{Pa}$ for the yield stress. The viscosity determined this way could be compared with theoretical considerations, for instance with the Krieger–Dougherty equation for hard spheres:

(2) $$\begin{array}{c} \frac{\eta_{eff}}{\eta_{0}}={\left(1-\frac{\phi_{p}}{\phi_{m}}\right)}^{-2.5\phi_{m}} \end{array}$$

Here, the effective viscosity $\eta_{eff}$ was correlated with the packing density of the particles $\phi_{p}$ and the maximum packing density $\phi_{m}$, which was assumed to be 0.6795 according to Farr et al. [24]. The viscosity of the carrier fluid was determined in a cone-plate rheometer with $\eta_{f}=0.53\mathrm{Pa}s$. For $\phi_{p}=0.42$, an apparent viscosity of $\eta_{eff}=2.72\mathrm{Pa}s$ was calculated, which was slightly below the measured data. The density of the mixture $\rho_{m}$ was dependent on the $\phi_{p}$, the density of the fluid $\rho_{l}$, and the particle density $\rho_{p}$. For the model concrete, $\rho_{m}=\phi_{p}\rho_{p}+\left(1-\phi_{p}\right)\rho_{f}=1717kg/m^{3}$.

In order to be able to compare different Bingham materials, it is useful to refer to dimensionless numbers. For flowing Bingham fluids, the Reynolds number Re, and the Hedstrom number He are the most suitable. The former describes the ratio of inertial to viscous forces. It is defined as:

(3) $$\begin{array}{c} Re=\frac{\rho_{m}\bar{v}D}{\eta_{eff}} \end{array}$$

with the density of the mixture $\rho_{m}$, the average velocity $\bar{v}$, the tube diameter *D*, and the effective viscosity $\eta_{eff}$. The Hedstrom number gives the ratio of the yield stress to the viscous stress in the Bingham fluid and is defined as:

(4) $$\begin{array}{c} He=\frac{D^{2}\rho_{m}\tau_{0}}{\eta_{eff}^{2}} \end{array}$$

As an example, the Reynolds and Hedstrom number is Re = 5.7 and He = 1.76, respectively, for a real self-compacting concrete (SCC) with material parameters $\rho =2300kg/m^{-3}$, η=24.1Pas and τ=31Pa [15], which is pumped with 0.5 m/s^−1^ through a pipeline of a diameter d=0.12m.

For the model concrete in this study, which was pumped with a constant velocity $\bar{v}=0.45m/s$^−1^ in the measuring pipe, a Reynolds number Re = 4.6 and a Hedstrom number He = 0.6 were obtained. Thus, both the Reynolds number and the Hedstrom number of our model system fit very well to those of the real concrete pumping process taken from the literature.

### 2.3. Experimental Procedure

The flow of the model concrete in the pipeline was pressure driven. For this purpose, a pulsating pressure $p_{0}$ was applied in the reservoir with the help of compressed air from a larger compressed air storage system. The pressure pulsations were tuned with the help of solenoid valves in the compressed air lines. As an example, Figure 2 gives the typical reservoir pressure history $p_{0}\left(t\right)$ for an experiment with the pre-run (loading) phase, the data acquisition period, and the post-run (discharge) phase.

**Figure 3:**
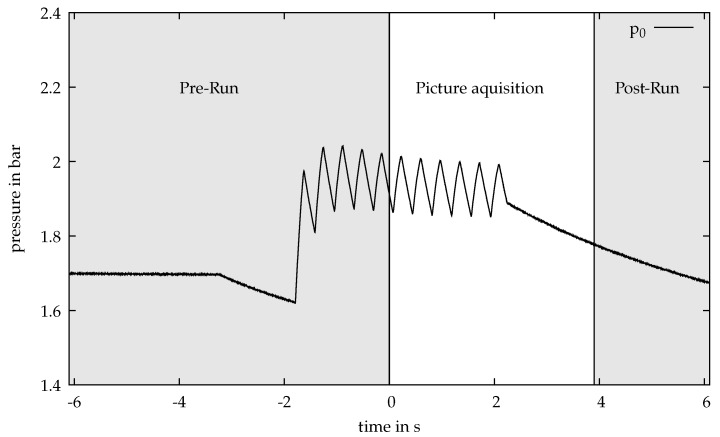
Reservoir pressure over time. The measurement with the camera starts at time t=0 s. The pressure during the pre-run phase, as well as during a post-run phase are recorded.

The results presented here were determined at a pulsating flow velocity. A mean pressure of 2 bar was set and the pressure oscillates with an amplitude of 0.2 bar and 2.5 Hz, as shown in Figure 3.

The pressure was initially built up in a buffer container to prevent later fluctuations in the system caused by an insufficient pressure reservoir. After the inlet pressure was reached, the solenoid valves were closed automatically. Then, the ball valve at the end of the measurement section was opened. As soon as the pressure drop was detected, the pulsation of the pressure started automatically.

The pressure was recorded during the entire measuring time. The optical recordings were started by a manual trigger. In addition, this trigger signal was also forwarded to the pressure measurement. In this way, pressure and speed could be correlated exactly. The measurement was started after five oscillations and recorded over another six oscillations. This ensured that the system had adapted to the pressure fluctuations. After the end of the oscillating regime, the flow was additionally recorded during linear conveying.

The image data obtained in this way were evaluated with the cross-correlation, and thus, the flow profiles resolved in time and space were generated. These in turn were correlated with the pressure data and used for the evaluation.

## 3. Results and Discussion

First of all, the velocities and pressure losses were measured to investigate the pumping process. Velocity data were extracted from optical investigations with PIV. The pressure loss was measured directly. Finally, the pressure loss would be determined from the measured rheological data for the model concrete and be compared with the measured data.

**Figure 4:**
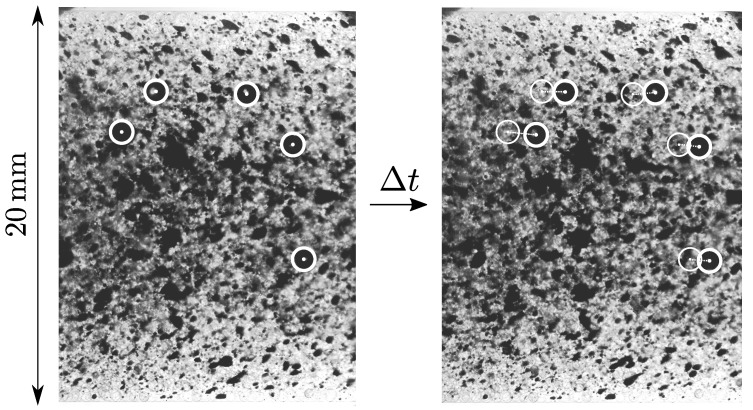
Two typical recordings from the measurements with a time difference of Δt=1.9. Easy to find structures like particles are marked. On the right, the displacements and the previous positions are shown with thin lines.

A typical picture taken by the camera is shown in Figure 4. This was adjusted for brightness and contrast during image processing. This processing was not necessary before the actual evaluation with PIV using the DAVIS 10 software from LaVision Göttingen, Germany). By comparing the displacement of patterns per time step, the speed could be calculated. In contrast to particle tracking, structures were tracked instead of individual particles. In the shown picture, particle displacements are shown for a clearer visualization.

By cross-correlating the individual images with known Δt, a velocity field was obtained for the entire image. It was assumed that the velocity did not change in the direction of the pipe axis, but only perpendicular to it. This allowed the averaging of the velocity vectors along the axis to increase the statistical accuracy. Thus, a velocity profile v(r) dependent on the distance from the pipe axis *r* was obtained.

**Figure 5:**
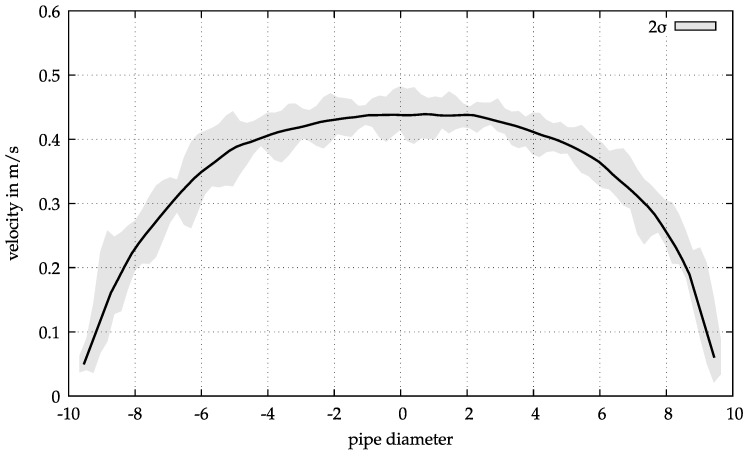
Velocity profile over the pipe diameter (smoothed). The statistical accuracy is shown in gray with twice the standard deviation. Gravity acts in the direction of the positive values of the diameter.

An example of such a velocity profile is shown in Figure 5. Here, the velocity profile in the constant velocity region towards the end of the measurements is shown. The statistical accuracy was described by twice the standard deviation.

A typical profile for a non-Newtonian fluid could be observed. The characteristic shear zone at the pipe wall is shown, as well as the zone of the plug-flow, from around −3 mm to 3 mm. The velocity at the pipe wall was not exactly zero here, as it would be with a classic Bingham fluid. Due to the presence of glass spheres directly at the wall, a finite velocity of about 0.05 m/s was still measured there. The classical no-slip condition did not apply to the granular material at this point.

Furthermore, a very slight asymmetry of the curve became apparent. It was assumed that this was caused by sedimentation of the glass spheres under gravity. Even if the high yield stress did not lead to segregation in the unsheared state, the yield stress was very well exceeded during pumping. As a result, the glass spheres could sediment, but much more slowly than in a fluid without a yield stress.

The advantage of the optical evaluation of the flow profile was that the profile was exactly known at any time of the measurement. This was a significant benefit, especially for non-constant flows, such as those that occur during the pumping of concrete, which could hardly be achieved by any other methods.

**Figure 6:**
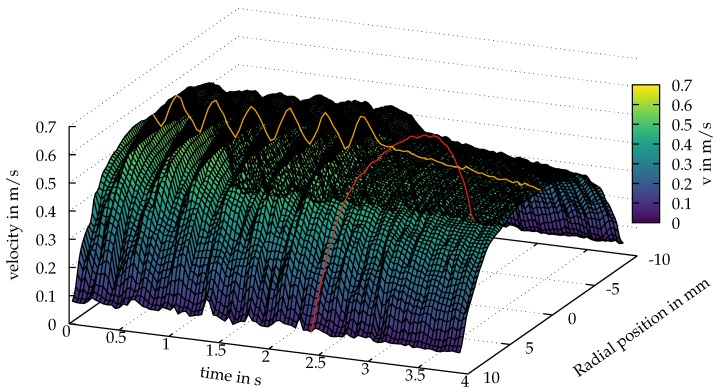
Velocity profiles during the pulsating flow over time. A single flow profile is shown in red, and the maximum velocity profile over time is shown in orange.

A three-dimensional representation of the flow profile of the measurement shown here can be found in Figure 6. There, the flow profiles are shown over a time period of 3.9 s, which corresponds to a total number of 2046 individual profiles.

From the flow profiles obtained in this way, the average flow velocity $\bar{v}\left(t\right)$ could be determined by integration over the pipe cross-section:

(5) $$\begin{array}{c} \bar{v}\left(t\right)=\frac{2}{R^{2}}\int_{0}^{R}r\;v\left(r,t\right)dr \end{array}$$

with the pipe radius *R*. Again, the average speed over the entire pumping process was thus known.

**Figure 7:**
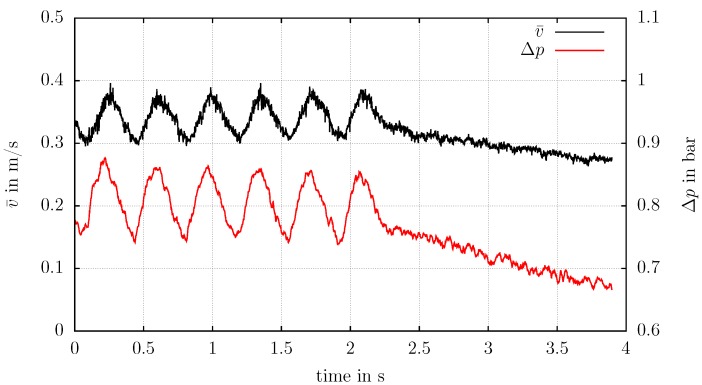
Average flow velocity $\bar{v}$ (black) and pressure loss Δp (red) over time.

In parallel to the optical measurements, the pressure loss Δp was recorded. In Figure 7, the pressure loss and average velocity are plotted over time.

For the dependence of the pressure loss on the flow velocity, the equation:

(6) $$\begin{array}{c} \Delta p=f\rho_{m}{\bar{v}}^{2}\frac{2L}{D} \end{array}$$

is used with the pipe diameter *D*, pipe length *L*, and the Fanning friction factor *f* [25]. In this case, the determination of *f* could be done without knowing the rheological parameters.

However, if the values for viscosity and yield stress were known, an analytical solution existed for the dependence of the pressure loss on the mean flow velocity, the so-called Buckingham–Reiner equation [26]:

(7) $$\begin{array}{c} Q=\bar{v}\;\cdot \;\pi R^{2}=\left\{\begin{array}{cc} 0 & \mathrm{for}\;\frac{\Pi R}{2\tau_{0}}\leq 1\\ \frac{\pi R^{4}\Pi }{8\eta_{eff}}\left(1-\frac{4}{3}\frac{2\tau_{0}}{\Pi R}+\frac{1}{3}{\left(\frac{2\tau_{0}}{\Pi R}\right)}^{4}\right) & \mathrm{for}\;\frac{\Pi R}{2\tau_{0}}\geq 1 \end{array}\right. \end{array}$$

with the flow rate *Q* and the reduced pressure loss Π=Δp/L over the pipe length *L*. The application of the Buckingham–Reiner equation on concrete was possible, although problematic due to the assumptions of the equation: a fully developed, laminar, and isothermal flow of an incompressible fluid in 1D was needed, which was fulfilled here; however the concrete was not homogeneous, and a slip layer (lubricating layer) was found, which made the application problematic; see [27]. Further, the equation was derived for steady flows.

Considering the above-introduced dimensionless ratios Re and He, the dimensionless Buckingham–Reiner equation:

(8) $$\begin{array}{c} f=\frac{16}{Re}\left(1+\frac{1}{6}\frac{He}{Re}-\frac{1}{3}\frac{He^{4}}{f^{3}Re^{7}}\right) \end{array}$$

with the Fanning friction factor *f* was obtained [28]. With that, the Fanning friction factor could be calculated from rheological data. For the model concrete, the needed parameters were known from rheological measurements.

**Figure 8:**
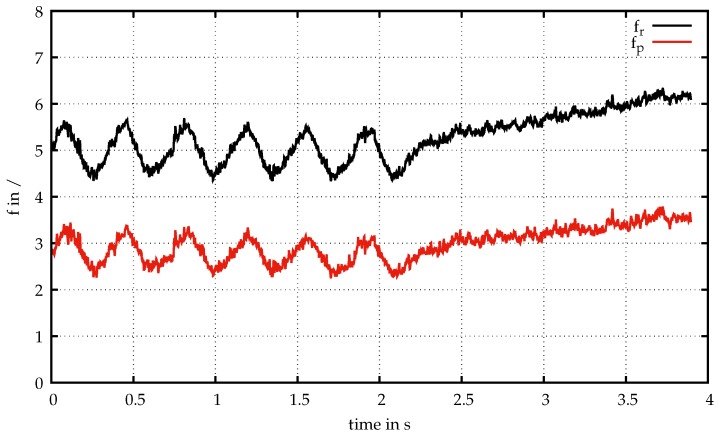
Fanning friction factor determined from the rheological data $f_{r}$ and from the pressure loss data $f_{p}$ over time.

For further discussion of the results in this paper, the friction factor from the calculation of the rheological data with Equation (8) is denoted as $f_{r}$, and the friction factor measured directly by pressure sensors and using Equation (6) is referred to as $f_{p}$. Figure 8 shows both factors for this measurement. In both cases, the curve followed the velocity pulsation. The dimensionless pressure losses were similar in each pulsation, and the sinusoidal form remained during the pumping process. Finally, in the linear pumping regime, $f_{r}$, as well as $f_{p}$ were increasing constantly.

**Figure 9:**
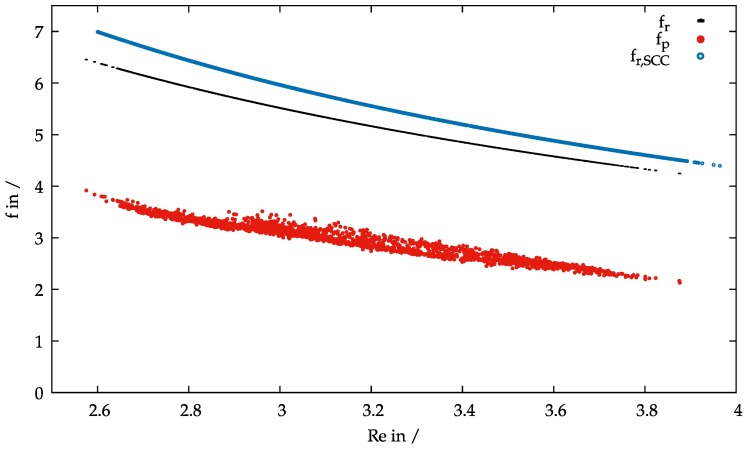
Fanning friction factor *f* plotted over Reynolds number Re for data from rheological measurements: $f_{r}$ and from pressure losses $f_{p}$. For comparison, $f_{r}$ for the rheological data from an SCCin the same Reynolds number range is plotted with the rheological data given in Section 2.

In addition to the time dependence, the friction factor is plotted against the Reynolds number in Figure 9. For $f_{r}$, this resulted in a clear functional relationship according to the Buckingham–Reiner Equation (8). There were no fluctuations in the system, since all rheological parameters were time and shear independent. If, however, the same was done for the data $f_{p}$ determined from the pressure losses, a temporally fluctuating pattern was obtained. This could be explained only by time or shear-dependent rheological parameters $\eta_{eff}\left(t,\dot{\gamma }\right)$ and $\tau_{0}\left(t,\dot{\gamma }\right)$. This in turn contradicted the assumption of a Bingham fluid. A precise analysis of this behavior will be the subject of future studies.

Furthermore, estimated values $f_{r}$ for the example SCCdescribed in Section 2 were inserted for comparison. Thereby, $f_{r}$ was also determined with the Buckingham–Reiner equation assuming constant viscosity and yield stress. An estimation in the given Reynolds number range was thus possible without performing a real pumping test with concrete.

The values for the model concrete and the real concrete showed a very good agreement. This indicated the suitability of the material as a model substance. Please note that the scaling to SCC was done by Hedstrom number He (rheology) and Reynolds number Re (flow rate) only. For real pumping experiments with SCC, effects such as the compressibility of real concrete by entrapped air and time-dependent rheological effects have to be considered, as well.

It was clearly noticeable that $f_{p}$ and thus the experimentally determined values were markedly lower than those resulting from the rheological data $f_{r}$. The pressure loss that was calculated from the rheological data thus overestimated the measured pressure loss in the system. The measured pressure loss was here reduced to around 60% during pumping. This behavior could be explained by the segregation of the particles on the pipe wall.

Under the assumption that a lubricating layer was formed, the pumping pressure, and thus $f_{p}$, decreased significantly. Due to the migration of the large particles into the pipe axis, the small particles and the fluid remained on the wall. Consequently, the friction with the wall would be reduced by the segregation. The viscosity near the wall would therefore decrease. At the same time, the packing density in plug flow was increased. However, only the rheological behavior at the pipe wall was relevant for the conveying pressure.

During the pumping of the model concrete, we could observe this segregation process. After a certain time of pumping, nearly no larger particle could be found any longer at the pipe wall. Thus, the packing density of the system was changing. According to Equation (2), this would result in a change in viscosity as well.

It should be noted that the model concrete could not represent all the properties of real concrete, and therefore, compromises must be made in favor of optical adaptation. For example, the carrier fluid and the particles were not of the same density, which could change the segregation properties. Likewise, non-spherical particles with a polydisperse distribution are present in real concrete, which could change the segregation behavior.

## 4. Conclusions

The behavior of a concrete under pulsating pumping conditions was investigated in the new experimental setup PulsaCoP. A transparent model concrete was employed, which allowed detailed analysis of velocity profiles and segregation phenomena in the concrete flow. Material and operation parameters were selected in order to meet the important dimensionless parameters of the Reynolds and Hedstrom number of real pumped concrete flows.

During the pumping experiments, pressure values were recorded at several locations along the pumping pipeline. Velocity data in the flow were obtained with the help of particle image velocimetry measurements in the model concrete. Pressure losses and mean flow velocities were correlated with respect to the Fanning friction factor and the Buckingham–Reiner equation, respectively. It was found that experimentally and semi-analytically obtained friction factors of the model concrete flow were in reasonable agreement. An estimation of friction factors in corresponding real-world flows of self-compacting concrete was given as well. Quantitative deviations between the different friction factors were caused among others by segregation and further particulate phenomena.

These effects are the subject of further investigations within the priority program Opus Fluidum Futurum of the German Research Foundation DFG. Among others, a more rigorous validation of the PulsaCoP experiment by comparison with data from large-scale pumping experiments with real concrete is planned. From these experiments, better correlations of the friction factors, segregation, concrete rheology, and concrete flow rates should be researched as well. Furthermore, these considerations are not limited to the flow in straight pipes, but will be applied to complex geometries in the future.
